# Pre-Stage Acute Kidney Injury Can Predict Mortality and Medical Costs in Hospitalized Patients

**DOI:** 10.1371/journal.pone.0167038

**Published:** 2016-12-01

**Authors:** Jeonghwan Lee, Seon Ha Baek, Shin Young Ahn, Ho Jun Chin, Ki Young Na, Dong-Wan Chae, Sejoong Kim

**Affiliations:** 1 Department of Internal Medicine, Hallym University Hangang Sacred Heart Hospital, Seoul, Korea; 2 Department of Internal Medicine, Seoul National University Bundang Hospital, Seongnam, Korea; 3 Department of Internal Medicine, Korea University Medical Center Korea University Guro Hospital, Seoul, Korea; Istituto Di Ricerche Farmacologiche Mario Negri, ITALY

## Abstract

The significance of minimal increases in serum creatinine below the levels indicative of the acute kidney injury (AKI) stage is not well established. We aimed to investigate the influence of pre-stage AKI (pre-AKI) on clinical outcomes. We enrolled a total of 21,261 patients who were admitted to the Seoul National University Bundang Hospital from January 1, 2013 to December 31, 2013. Pre-AKI was defined as a 25–50% increase in peak serum creatinine levels from baseline levels during the hospital stay. In total, 5.4% of the patients had pre-AKI during admission. The patients with pre-AKI were predominantly female (55.0%) and had a lower body weight and lower baseline levels of serum creatinine (0.63 ± 0.18 mg/dl) than the patients with AKI and the patients without AKI (P < 0.001). The patients with pre-AKI had a higher prevalence of diabetes mellitus (25.1%) and malignancy (32.6%). The adjusted hazard ratio of in-hospital mortality for pre-AKI was 2.112 [95% confidence interval (CI), 1.143 to 3.903]. In addition, patients with pre-AKI had an increased length of stay (7.7 ± 9.7 days in patients without AKI, 11.4 ± 11.4 days in patients with pre-AKI, P < 0.001) and increased medical costs (4,061 ± 4,318 USD in patients without AKI, 4,966 ± 5,099 USD in patients with pre-AKI, P < 0.001) during admission. The adjusted hazard ratio of all-cause mortality for pre-AKI during the follow-up period of 2.0 ± 0.6 years was 1.473 (95% CI, 1.228 to 1.684). Although the adjusted hazard ratio of pre-AKI for overall mortality was not significant among the patients admitted to the surgery department or who underwent surgery, pre-AKI was significantly associated with mortality among the non-surgical patients (adjusted HR 1.542 [95% CI, 1.330 to 1.787]) and the patients admitted to the medical department (adjusted HR 1.384 [95% CI, 1.153 to 1.662]). Pre-AKI is associated with increased mortality, longer hospital stay, and increased medical costs during admission. More attention should be paid to the clinical significance of pre-AKI.

## Introduction

Acute kidney injury (AKI) is a common complication among hospitalized patients with acute illness or undergoing major surgery. The incidence of in-hospital AKI is approximately 15–20%, and recent studies have suggested that the AKI incidence is steadily increasing [1,2]. AKI worsens patients’ clinical outcomes, with increased mortality; the in-hospital mortality of patients with AKI is reported to be up to 25% [3]. In addition, AKI is associated with increased length of hospital stay, medical costs, post-discharge chronic kidney disease and end-stage renal disease [4,5].

According to the 2012 Kidney Disease Improving Global Outcomes (KDIGO) guidelines, AKI is defined as an increase in the serum creatinine level of 0.3 mg/dl within 48 hours or up to 1.5 times the baseline level within the prior 7 days [6]. Although these guidelines were developed through the explicit process of evidence review and appraisal, the cut-off value for AKI is somewhat arbitrary. In clinical practice, clinicians frequently encounter patients with mild increases in serum creatinine levels below the diagnostic criteria for AKI. However, the significance of minimal increases in serum creatinine below the levels indicative of AKI is not well established. Most clinicians do not pay serious attention to small incremental changes in serum creatinine among hospitalized patients, which can often be attributed to normal laboratory variations. Previous reports stated that mild increases in serum creatinine are associated with the development of AKI, increased mortality, and adverse outcomes [7–9]. However, these investigations were limited to particular patient groups, such as patients with heart failure or patients who underwent cardiac surgery, which limits the generalization of the results. Therefore, investigating the incidence and prognosis of pre-AKI among unselected hospitalized patients is important. In addition, in patients with low body muscle mass, including Asians, mild increases in serum creatinine might be clinically more important than in other patients. We aimed to investigate the influence of pre-AKI on clinical outcomes among hospitalized patients.

## Materials and Methods

### Patients and data collection

This study enrolled a total of 21,261 adult patients (aged ≥ 18 years) who were admitted to the Seoul National University Bundang Hospital (SNUBH) from January 2013 to December 2013. The Institutional Review Board of the Seoul National University Hospital approved this investigation (No. B-1511/322-114). All clinical and laboratory data were routinely stored in the electronic medical database and the SNUBH Clinical Data Warehouse (SNUBH-CDW), and retrieved for the academic purpose after the approval of institutional review board. Parts of this observational cohort have already been described in previous publication [10]. Informed consents were waived due to the retrospective study design. We screened all adult patients admitted to the hospital over a 1-year period and excluded patients whose serum creatinine levels were not tested at least twice during admission. Among the 21,573 admission patients, 312 patients who were on maintenance dialysis or who underwent kidney transplantation were excluded. Baseline creatinine levels were defined as follows: 1) the lowest serum creatinine value during the 3 months prior to admission; 2) if no data were available for criteria 1, the lowest serum creatinine value between 3–6 months prior to admission; and 3) if no data were available for criteria 2, the serum creatinine level derived from the estimated glomerular filtration rate (GFR; 75 ml/min/1.73 m^2^) [6,11,12]. The AKI stages were classified according to the 2012 KDIGO criteria using only the serum creatinine value [6]. In addition, pre-stage AKI (pre-AKI) was defined as an increase in serum creatinine levels of 25–50% from the baseline value. Laboratory data aside from the serum creatinine levels were obtained from the first laboratory data recorded during admission. The GFR was estimated using the MDRD equation and the Korean coefficient of MDRD [13,14]. Data on comorbidities, including hypertension, diabetes mellitus, angina, ischemic heart disease, myocardial infarction, heart failure, cerebrovascular disease, and malignancy, were based on the registered list of diagnoses in the electronic medical records database just before admission. Additionally, information concerning diabetes or hypertension medications was used to diagnose the presence of comorbid diabetes mellitus and hypertension. The comorbidity scores were calculated as the sum of the number of comorbidities. The purpose of admission was classified as either surgical admission (surgery was performed during the admission) or medical admission (surgery was not performed during the admission). The department of admission was classified as the medical department (department of internal medicine, geriatrics, neurology, pediatrics, and psychology), surgical department (department of general surgery, neurosurgery, chest surgery, spine surgery, plastic surgery, orthopedics, obstetrics and gynecology, otorhinolaryngology, ophthalmology, dentistry, and urology), combined department (cancer center, cranio-neurological center, cardiovascular center, joint and rheumatology center, respiratory center, and department of emergency), and others (department of anesthesiology, dermatology, diagnostic radiology, nuclear medicine, rehabilitation, and clinical trial centers). The date of mortality was obtained from hospital medical records and the national government’s death database prior to August 31, 2015. Information on renal replacement therapy and status of end-stage renal disease during the 1 year after hospital discharge was collected using the end-stage renal disease registry of the Korean Society of Nephrology (KSN) [15–17]. Medical expenditures were evaluated based on direct medical costs, including physician costs, admission costs, surgery costs, therapy costs, and medicine costs. The medical costs were calculated in Korean Won (KRW) (US$1 = 1,100 KRW in 2015).

### Statistical analysis

All calculations and statistical analyses were performed using the IBM SPSS Statistics 22 software (IBM, Armonk, NY, USA). Continuous variables were expressed as the mean and standard deviation, and categorical variables were described numerically using a percentage. All of the variables were tested for normality using the Q-Q plot and the Kolmogorov-Smirnov test. Comparisons between the groups based on the stages of AKI were performed using a one-way ANOVA (analysis of variance) for continuous variables and the Chi-square or Fisher’s exact tests for categorical variables when appropriate. Patient survival was compared using the Kaplan-Meier survival curve and a log-rank test. The hazard ratios (HRs) and 95% confidence intervals (CIs) of the clinical parameters for in-hospital mortality were calculated using a univariate and multivariate Cox proportional hazard model analysis. The covariates included in the multivariate analysis were selected based on clinical significance and included age, sex, body mass index, surgery, comorbidity scores, admission to an intensive care unit, hemoglobin levels, and albumin levels. In the analysis of medical costs, comparisons between groups based on the stages of AKI were performed using a one-way ANOVA and an ANCOVA. In the ANCOVA analysis, factors that demonstrated a significant effect on total medical costs, including the duration of admission, age, sex, body mass index, comorbidities, admission to an intensive care unit, and surgery, were included for adjustment. All of the statistical tests were 2-tailed. A *P*-value < 0.05 was considered statistically significant.

## Results

### Patient characteristics according to the acute kidney injury stage

A total of 21,261 patients were enrolled in this study. The clinical and laboratory characteristics according to the stages of AKI are presented in Table 1. The mean age of the patients was 58.7 ± 17.1 years old. Just over half (52.6%) of the patients were male. The baseline creatinine levels were 0.90 ± 0.47 mg/dl, and the estimated GFR was 86.2 ± 35.4 ml/min/1.73 m^2^. A total of 7,691 (36.2%) patients underwent surgery during admission, and 2,978 (14.0%) patients were admitted to the intensive care unit. Of those patients who were admitted to the intensive care unit, 52.5% were admitted to the surgical intensive care unit, 24.0% to the neurological unit, 15.5% to the medical unit, and 8.0% were emergent.

**Table 1:** Patient Characteristics According to the Acute Kidney Injury Stage.

Variables	Total (n, 21261)	No AKI (n, 17801, 83.7%)	Pre-AKI (n, 1139, 5.4%)	AKI (n, 2321, 10.9%)	P*
Age, years (n, 21261)	58.7 ± 17.1	57.5 ± 17.0	60.9 ± 16.9	66.6 ± 15.8	< 0.001
Sex, male (n, 21261)	11193 (52.6%)	9368 (52.6%)	513 (45.0%)	1312 (56.5%)	< 0.001
Height, cm (n, 19471)	162.3 ± 9.2	162.5 ± 9.1	160.9 ± 9.3	161.8 ± 9.1	< 0.001
Body weight, kg (n, 19498)	62.8 ± 12.2	63.2 ± 12.1	60.3 ± 11.9	61.1 ± 12.9	< 0.001
Body mass index (n, 19255)	23.8 ±3.7	23.9 ± 3.7	23.3 ± 3.8	23.3 ± 4.0	< 0.001
Creatinine, mg/dl (n, 21261)	0.90 ± 0.47	0.90 ± 0.36	0.63 ± 0.18	0.99 ± 0.98	< 0.001
GFR, ml/min/1.73m^2^ (n, 21261)	86.2 ± 35.4	82.1 ± 23.7	122.7 ± 44.0	99.8 ± 72.7	< 0.001
Hemoglobin, g/dl (n, 20897)	12.6 ± 2.1	12.8 ± 2.0	12.0 ± 2.1	11.6 ± 2.4	< 0.001
Albumin, mg/dl (n, 20821)	3.9 ± 0.6	4.0 ± 0.6	3.7 ± 0.6	3.6 ± 0.6	< 0.001
CRP, mg/l (n, 11807)	5.31 ± 6.29	5.10 ± 6.09	4.78 ± 5.95	6.72 ± 7.30	< 0.001
Glucose, mg/dl (n, 18599)	128.5 ± 57.5	125.1 ± 52.2	129.2 ± 53.4	151.6 ± 83.5	< 0.001
Cholesterol, mg/dl (n, 20676)	170.2 ± 47.5	172.4 ± 45.9	164.2 ± 46.6	156.1 ± 56.2	< 0.001
Surgery vs. Medical (n, 21261)	7691 (36.2%)	6587 (37.0%)	373 (32.7%)	731 (31.5%)	< 0.001
Department (n, 21261)					< 0.001
Medical (n, 5248)	5248 (24.7%)	3982 (22.4%)	413 (36.3%)	853 (36.8%)	
Surgical (n, 6123)	6123 (28.8%)	5445 (30.6%)	243 (21.3%)	435 (18.7%)	
Combined (n, 9619)	9619 (45.2%)	8130 (45.7%)	473 (41.5%)	1016 (43.8%)	
Others (n, 271)	271 (1.3%)	244 (1.4%)	10 (0.9%)	17 (0.7%)	
Comorbidity (n, 21261)					
Angina (n, 21261)	882 (4.1%)	799 (4.5%)	29 (2.5%)	54 (2.3%)	< 0.001
Myocardial infarction (n, 21261)	406 (1.9%)	312 (1.8%)	14 (1.2%)	80 (3.4%)	< 0.001
Ischemic heart disease (n, 21261)	568 (2.7%)	466 (2.6%)	20 (1.8%)	82 (14.4%)	0.005
Heart failure (n, 21261)	236 (1.1%)	109 (0.6%)	22 (1.9%)	105 (4.5%)	< 0.001
Cerebrovascular disease (n, 21261)	1271 (6.0%)	1001 (5.6%)	74 (6.5%)	196 (8.4%)	< 0.001
Hypertension (n, 21261)	3985 (18.7%)	2988 (16.8%)	265 (23.3%)	722 (31.1%)	< 0.001
Malignancy (n, 21261)	5362 (25.2%)	4325 (24.3%)	371 (32.6%)	666 (28.7%)	< 0.001
Diabetes mellitus (n, 21261)	4610 (21.7%)	3304 (18.6%)	286 (25.1%)	1020 (22.1%)	< 0.001
Comorbidity Score (n, 21261)					< 0.001
0	9410 (44.3%)	8457 (47.5%)	417 (36.6%)	536 (23.1%)	
1	7500 (35.3%)	6133 (34.5%)	429 (37.7%)	938 (40.4%)	
≥ 2	4351 (20.5%)	3211 (18.0%)	293 (25.7%)	847 (36.5%)	
ICU (n, 21261)	2978 (14.0%)	2133 (12.0%)	152 (13.3%)	694 (29.9%)	< 0.001
ICU types (n, 1704)					< 0.001
Medical	264 (15.5%)	109 (10.2%)	15 (15.2%)	140 (26.3%)	
Surgical	895 (52.5%)	570 (53.1%)	59 (59.6%)	266 (50.0%)	
Neurological	409 (24.0%)	329 (30.7%)	21 (21.2%)	59 (11.1%)	
Emergency	136 (8.0%)	65 (6.1%)	4 (4.0%)	67 (12.6%)	

Abbreviations: AKI, acute kidney injury; CRP, C-reactive protein; ICU, intensive care units

*P, comparing patients with no AKI, pre-AKI, and AKIs

Of the 21,216 admissions, 2,321 patients (10.9%) had AKI, including 1,578 (3.7%) classified as AKI stage 1, 410 (1.9%) classified as AKI stage 2, and 333 (1.6%) classified as AKI stage 3 (Table 1). Pre-AKI was discovered in 1,139 patients (5.4%). The patients with pre-AKI were predominantly female (55.0%). The patients with pre-AKI had smaller body weights compared to both the patients without AKI and the patients with AKI. The baseline serum creatinine levels were lower among the pre-AKI patients than the patients without AKI and the patients with AKI. The patients with pre-AKI had a higher prevalence of diabetes mellitus (25.1%) and malignancy (32.6%) than the patients without AKI and the patients with AKI.

### Patient survival according to the acute kidney injury stage

**Fig 1:**
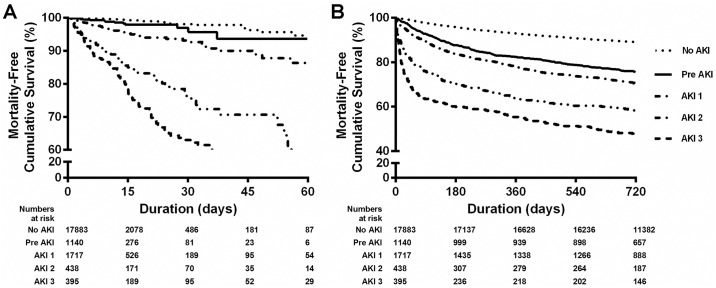
Survival according to the acute kidney injury (AKI) stage. (A) Death-free survival during admission. Patient survival decreased as the AKI stage increased, including pre-stage AKI (log-rank, P < 0.001). (B) Death-free survival during the follow-up. Patient survival decreased as the AKI stage increased, including pre-stage AKI (log-rank, P < 0.001).

The death-free survival of the patients during admission is presented in Fig 1A. Patient survival deteriorated with more advanced stages of AKI (P < 0.001). During the 8.9 ± 12.7 days of admission, 321 deaths occurred. The patient clinical outcomes according to the stages of AKI are summarized in S1 Table. The in-hospital mortality of the patients without AKI, with pre-AKI, and with stage 1, stage 2, and stage 3 AKI was 0.3, 1.3, 4.4, 17.1, and 32.4%, respectively (P < 0.001). The causes of in-hospital mortality did not differ among the stages of AKI. Table 2 summarizes the hazard ratio of pre-AKI on in-hospital mortality after adjusting for age, sex, body mass index, comorbidity scores, admission to intensive care units, department of admission, hemoglobin levels, and serum albumin levels. The adjusted hazard ratio of the in-hospital mortality for pre-AKI was 2.112 (95% CI, 1.143 to 3.903). Among the patients who did not undergo surgery or those who were admitted to the medical department, the adjusted hazard ratio of pre-AKI for in-hospital mortality was 2.277 (95% CI, 1.195 to 4.339) and 2.473 (95% CI, 1.022 to 5.986), respectively.

**Table 2:** Pre-Stage Acute Kidney Injury and All-Cause and Cause-Specific In-Hospital Mortality.

Cause of Death	Cases Per Person-Year	Incidence Density*	aHR^†^ (95% CI)
All cause			
All participants (n = 21261)	321/519.2	61820.1	2.112 (1.143 to 3.903)
Surgery (n = 7691)	37/243.9	15170.3	0.763 (0.092 to 6.313)
Non-surgery (n = 13570)	284/275.4	103141.0	2.277 (1.195 to 4.339)
Department, medicine (n = 5248)	130/134.8	96424.7	2.473 (1.022 to 5.986)
Department, surgery (n = 6123)	27/145.7	18530.1	7.059 (0.409 to 121.850)
Department, combined (n = 9619)	164/233.6	70213.3	1.565 (0.605 to 4.050)
Department, others (n = 271)	no mortality		
Comorbidities, diabetes (n = 4610)	180/160.5	112153.7	1.239 (0.371 to 4.142)
Comorbidities, hypertension (n = 3985)	100/104.0	96151.6	3.201 (1.004 to 10.209)
Cause of death			
Cancer	63/519.2	12132.9	1.018 (0.293 to 3.535)
Cardiovascular	67/519.2	12903.3	4.961 (1.263 to 19.490)
Infections	91/519.2	17525.3	1.739 (0.498 to 6.078)
Bleeding	20/519.2	3851.7	3.180 (0.324 to 31.204)
Others	80/519.2	15406.9	2.543 (0.698 to 9.271)

Abbreviations: aHR, adjusted hazard ratio; 95% CI, 95% confidence interval

*Incidence density (per 100,000 person-year).

^†^aHR and 95% CI of the participants with pre-AKI compared to the patients without pre-AKI and the patients with AKIs as determined by the Cox proportional hazards model adjusted for age, sex, body mass index, comorbidity scores, admission to intensive care units, department of admission, hemoglobin levels, and serum albumin levels.

During the mean follow-up of 2.0 ± 0.6 years, 3,182 deaths occurred (Fig 1B, Table 3). The adjusted hazard ratio of all-cause mortality for pre-AKI was 1.473 (95% CI, 1.228 to 1.684). Although the adjusted hazard ratio of pre-AKI for overall mortality was not significant among patients admitted to the surgery department or undergoing surgery, pre-AKI was significantly associated with mortality among the non-surgical patients (adjusted HR 1.542 [95% CI, 1.330 to 1.787]) and the patients admitted to the medical department (adjusted HR 1.384 [95% CI, 1.153 to 1.662]).

**Table 3:** Pre-Stage Acute Kidney Injury and Long-Term Mortality.

Subgroups	Cases Per Person-Year	Incidence Density*	aHR^†^ (95% CI)
All cause			
All participants (n = 21261)	3182/41728.5	7625.5	1.473 (1.288 to 1.684)
Male participants (n = 11193)	1968/21533.7	9139.2	1.486 (1.246 to 1.773)
Female participants (n = 10068)	1214/20194.9	6011.4	1.449 (1.179 to 1.782)
Age, < 35 (n = 2383)	62/5055.4	1226.4	2.630 (1.001 to 6.909)
Age, 35–45 (n = 2242)	125/4674.1	2674.3	1.938 (0.954 to 3.939)
Age, 45–55 (n = 3365)	321/6897.0	4654.2	2.049 (1.416 to 2.964)
Age, 55–65 (n = 4204)	547/8376.7	6530.0	1.938 (1.452 to 2.588)
Age, ≥ 65 (n = 9067)	2127/16725.3	12717.2	1.224 (1.026 to 1.461)
BMI, < 18.5 (n = 1263)	468/2001.6	23381.0	1.011 (0.735 to 1.390)
BMI, 18.5–25 (n = 11213)	1843/21621.9	8523.8	1.561 (1.320 to 1.846)
BMI, 25–30 (n = 5864)	538/11975.6	4492.5	1.791 (1.296 to 2.474)
BMI, 30–35 (n = 799)	45/1661.4	2708.6	3.017 (1.021 to 8.915)
BMI, ≥ 35 (n = 116)	6/238.0	2520.8	ND^‡^, *P* = 0.997
Surgery (n = 7691)	730/15838.3	4609.1	1.068 (0.773 to 1.475)
Non-surgery (n = 13570)	2452/25890.2	9470.8	1.542 (1.330 to 1.787)
Department, medicine (n = 5248)	1528/9193.1	16621.1	1.384 (1.153 to 1.662)
Department, surgery (n = 6123)	502/12682.4	3958.2	1.287 (0.897 to 1.847)
Department, combined (n = 9619)	1139/19275.8	5909.0	1.545 (1.220 to 1.958)
Department, others (n = 271)	13/577.2	2252.1	ND^‡^, *P* = 0.987
Comorbidities, diabetes (n = 4610)	1212/8251.1	14689.0	1.456 (1.146 to 1.850)
Comorbidities, hypertension (n = 3985)	735/7492.8	9809.4	1.806 (1.377 to 2.368)
ICU (n = 2978)	611/5504.2	11100.6	1.496 (1.032 to 2.168)

Abbreviations: aHR, adjusted hazard ratio; BMI, body mass index; ICU, intensive care unit; ND, not determined; 95% CI, 95% confidence interval

*Incidence density (per 100,000 person-year).

^†^aHR and 95%CI of the participants with pre-AKI compared to the patients without pre-AKI and the patients with AKI as determined by the Cox proportional hazards model adjusted for age, sex, body mass index, comorbidity scores, admission of intensive care units, department of admission, hemoglobin levels, and serum albumin levels.

^‡^ND is the undetermined adjusted hazard ratio due to the low numbers of participants in each subgroup (eg. aHR = 0.000, 95% CI = 0.000 to ∞).

### Patient outcomes and medical costs according to the acute kidney injury stage

**Fig 2:**
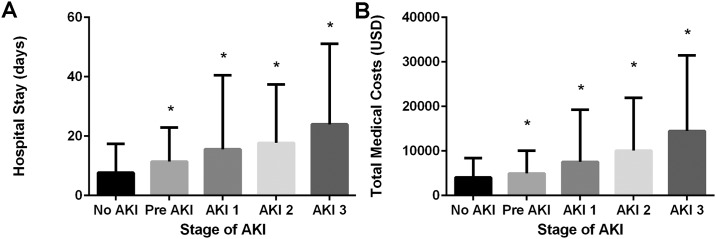
Outcomes of pre-stage acute kidney injury (AKI). (A) The duration of admission in patients with pre-stage AKI was significantly longer than in patients without AKI. (B) Total medical costs according to the stages of AKI are presented. The total medical costs gradually increased as the stage of AKI, including pre-AKI, increased (P < 0.001). *, significant difference (P < 0.05) compared to the patients without AKI.

The duration of total admission and intensive care unit admission increased according to the AKI stage, including pre-AKI (S1 Table). The duration of admission was significantly longer in the patients with pre-AKI than in the patients without AKI (Fig 2A). The prevalence of renal replacement therapy, including hemodialysis and hemodiafiltration, in the intensive care units and post-discharge end-stage renal disease were not significantly different between the patients without AKI and the patients with pre-AKI (S1 Table). A comparison of total medical costs according to the stage of AKI and other clinical variables is presented in S2 Table and Fig 2B. The total medical costs of the patients without AKI were 4,060.5 ± 4,318.3 USD. The total medical costs of the patients with pre-AKI and the patients with stage 1, stage 2, and stage 3 AKI were 4,965.5 ± 5,098.8 USD, 7,554.8 ± 11,726.7 USD, 10,085.4 ± 11,850.3 USD, and 14,474.3 ± 16,970.8 USD, respectively. The total medical costs gradually increased according to the stages of AKI, including pre-AKI (P < 0.001). After adjusting for the duration of admission, age, sex, body mass index, comorbidities, admission to ICU, and operation (surgery), a multivariate analysis revealed that the total medical costs of the patients with pre-AKI were significantly higher than the costs of the patients without AKI (S2 Table).

## Discussion

Using a large-scale cohort of patients admitted to a single tertiary university hospital, we demonstrated that pre-AKI, which was defined as a mild increase (25–50%) in serum creatinine from the baseline level, was associated with increased mortality, longer hospitalization, and increased medical costs. The patients with pre-AKI were predominantly female and had a lower body weight, lower serum creatinine level, and higher GFR. The patients with pre-AKI also had a higher prevalence of diabetes mellitus (25.1%) and malignancy (32.6%), and the patients who were admitted to the surgical intensive care unit had a higher prevalence of pre-AKI. Although pre-AKI was not associated with in-hospital and long-term mortality among the patients who had undergone surgery or who were admitted to the surgery department, pre-AKI was associated with increased mortality among the patients who were admitted to the medical department.

AKI has an adverse effect on hospitalized patient outcomes. In-hospital mortality is reported to be 15–30% in patients with AKI compared to general hospitalized patients [2]. Increased length of hospital stay and increased medical costs are other adverse outcomes associated with AKI [18]. Recently, studies have reported that patients with AKI have poorer outcomes after hospital discharge, including increased long-term mortality, increased medical costs, and development of chronic kidney disease and end-stage kidney disease [4,19,20]. However, the significance of minimal increases in serum creatinine below the levels indicative of AKI is not well established.

Several studies have investigated the clinical significance of a mild increase in serum creatinine levels. Gottlieb et al. reported that subtle increases in serum creatinine (approximately 0.1 mg/dl) were associated with increased mortality and an increased length of hospitalization in heart failure patients [9]. Weisbord et al. showed that small absolute (0.25 to 0.5 mg/dl) and relative (25–50%) increases in serum creatinine were significantly associated with in-hospital mortality among patients who underwent coronary angiography [21]. Metra et al. found that impaired renal function, as measured by a greater than 25% increase in serum creatinine levels from baseline, was associated with mortality and re-hospitalization in heart failure patients [22]. Newsome et al. reported that minimal increases in serum creatinine (approximately 0.1 mg/dl) were associated with increased long-term mortality and end-stage renal disease in patients with acute myocardial infarction [23]. Recently, Liotta et al. showed that small increases in serum creatinine (0–0.3 mg/dl) were significantly associated with long-term mortality, but not with short-term (1 month) mortality, in patients following cardiac surgery [24]. Kork et al. revealed that minimal increases in serum creatinine (25–50% increase from baseline, value less than 0.3 mg/dl) resulted in increased in-hospital mortality and length of hospital stay in post-surgical patients [25]. Although these studies investigated the effect of small increases in serum creatinine on clinical outcomes, they are limited to specific patient populations, i.e., patients who underwent cardiac surgery or heart failure patients. In this study, we enrolled all hospitalized patients during a 1-year period and investigated the effect of pre-AKI on short-term and long-term mortality, length of hospital stay, and medical costs. In addition, it is important to note that pre-AKI was significantly associated with mortality among non-surgical patients admitted to the medical department.

In this study, the range of mild increases in serum creatinine (approximately 0.1–0.29 mg/dl) was below the levels indicative of the different AKI stages. The reference change value of serum creatinine, which might reflect normal laboratory analytic variability and biological within-individual variability, is estimated at 14–17% [26]. Therefore, small increases in serum creatinine might reflect normal variability in serum creatinine within the reference range. However, in patients with low baseline serum creatinine values, small increases in serum creatinine (approximately 0.1–0.29 mg/dl) can constitute a significant increase of more than 25% in the serum creatinine level from the baseline value. In fact, low dietary protein intake and reduced creatinine generation from lower muscle mass (due to old age, female gender, muscle wasting conditions, amputation, malnutrition, and critical illness) can contribute to lower levels of serum creatinine than the general population. In this study, patients with pre-AKI were predominantly female and had a lower body weight, lower serum creatinine level, and a higher GFR. In addition, patients with pre-AKI had a higher prevalence of diabetes mellitus and malignancy. Therefore, among patients who have a lower muscle mass and lower baseline serum creatinine level (e.g., Asians, females, or patients with a chronic illness), small increases in serum creatinine might be more important than general expectations. Clinicians should not neglect these mild increases in serum creatinine among hospitalized patients and should immediately refer these patients to specialists or nephrologists. Unrevealed causes of pre-stage AKI should be identified and quickly corrected to prevent adverse outcomes.

This study utilized a large-scale cohort of patients admitted to a single university hospital over a 1-year period. Although we found unique characteristics of pre-stage AKI and a poor clinical impact on various outcomes, this study has several limitations. First, this study was conducted in a single hospital, and almost all of the patients were Korean. These clinical settings limit the generalizability of the study results to other hospital settings and other races. Second, estimating baseline serum creatinine values from the back-calculation of the estimated MDRD GFR cannot be as exact as the general expectations. Although a single imputation method was validated and recommended in several guidelines [6,11,12], this method could overestimate the incidence of AKI [27]. Finally, due to the retrospective cohort study design, we were unable to determine the exact causes of pre-AKI that influenced the poor clinical outcomes. However, this study still has clinical impact because of the large number of participants and long-term follow-up.

Pre-AKI was associated with increased mortality, longer length of hospital stay, and increased medical costs. In addition, pre-AKI was prevalent among female patients with low serum creatinine levels, low body weight, diabetes, or malignancies. Therefore, the clinical significance of pre-AKI might be more important among patients with low body muscle mass, particularly Asians or patients with chronic illnesses. Pre-stage AKI was revealed to have a more deleterious impact on clinical outcomes among patients admitted to medical departments or patients who had not undergone surgery during hospitalization. Clinicians should be aware of the higher incidence and poorer clinical outcomes of pre-stage AKI among patients with these clinical characteristics. The progression of pre-AKI and its deleterious effects could be prevented through the prompt identification of the unrevealed causes of pre-AKI and proper management, including adequate volume control, removal of nephrotoxic drugs, and correction of other causative factors of pre-AKI. In future studies, the impact of early identification and intervention for pre-AKI on the clinical outcomes should be evaluated.

## Supporting Information

S1 Table Clinical Outcomes According to the Acute Kidney Injury Stage. (DOCX)

S2 Table Total Medical Costs according to the Acute Kidney Injury Stages and Other Clinical Factors. (DOCX)

S1 Dataset Dataset Used in the Analysis. (XLSX)
