# Supplementary material for: Pre-Stage Acute Kidney Injury Can Predict Mortality and Medical Costs in Hospitalized Patients
Source: PLoS One. 2016 Dec 1;11(12):e0167038. doi: 10.1371/journal.pone.0167038 (PMC5132306; doi:10.1371/journal.pone.0167038)
Supplement: S1 Table — (DOCX) [file pone.0167038.s001.docx]

S1 Table. Clinical Outcomes According to the Acute Kidney Injury Stage

| Variables | Total (n, 21261) | No AKI (n, 17801, 83.7%) | Pre-AKI (n, 1139, 5.4%) | AKI (n, 2321, 10.9%) | *P*^*^ |
| --- | --- | --- | --- | --- | --- |
| In-hospital mortality | 321 (1.5%) | 58 (0.3%) | 15 (1.3%) | 248 (10.7%) | < 0.001 |
| Causes |  |  |  |  | 0.797 |
| Bleeding | 20 (6.2%) | 3 (5.2%) | 1 (6.7%) | 16 (6.5%) |  |
| Malignancy | 63 (19.6%) | 16 (27.6%) | 4 (26.7%) | 43 (17.3%) |  |
| Cardiovascular | 67 (20.9%) | 9 (15.5%) | 3 (20.0%) | 55 (22.2%) |  |
| Infection | 91 (28.3%) | 17 (29.3%) | 3 (20.0%) | 71 (28.6%) |  |
| Others | 80 (24.9%) | 13 (22.4%) | 4 (26.7%) | 63 (25.4%) |  |
| Duration of admission, days | 8.9 ± 12.7 | 7.7 ± 9.7 | 11.4 ± 11.4 | 17.2 ± 24.6 | < 0.001 |
| Duration of ICU, days | 2.5 ± 7.9 | 1.8 ± 3.8 | 2.0 ± 2.7 | 4.7 ± 14.6 | < 0.001 |
| Costs, USD | 4647.7 ± 6116.2 | 4060.5 ± 4318.3 | 4965.5 ± 5098.8 | 8994.6 ± 12859.9 | < 0.001 |
| HD | 105 (0.5%) | 9 (0.1%) | 0 (0.0%) | 96 (4.1%) | < 0.001 |
| CRRT | 127 (0.6%) | 9 (0.0%) | 1 (0.1%) | 119 (5.1%) | < 0.001 |
| Post-discharge ESRD | 163 (0.8%) | 40 (0.2%) | 1 (0.1%) | 122 (5.3%) | < 0.001 |

Abbreviations: AKI, acute kidney injury; CRRT, continuous renal replacement therapy; HD, hemodialysis; ICU, intensive care units

^*^*P*, comparing patients with no AKI, pre-AKI, and AKIs
