# Supplementary material for: Pre-Stage Acute Kidney Injury Can Predict Mortality and Medical Costs in Hospitalized Patients
Source: PLoS One. 2016 Dec 1;11(12):e0167038. doi: 10.1371/journal.pone.0167038 (PMC5132306; doi:10.1371/journal.pone.0167038)
Supplement: S2 Table — (DOCX) [file pone.0167038.s002.docx]

S2 Table. Total Medical Costs according to the Acute Kidney Injury Stages and Other Clinical Factors

| Variables | Total medical costs | P^1^ (ANOVA) | P^2^ (ANCOVA) |
| --- | --- | --- | --- |
| Stages of AKI |  | < 0.001 | < 0.001 |
| No AKI | 4060.5 ± 4318.3 |  |  |
| Pre-AKI | 4965.5 ± 5098.8 |  |  |
| AKI stage 1 | 7554.8 ± 11726.7 |  |  |
| AKI stage 2 | 10085.4 ± 11850.3 |  |  |
| AKI stage 3 | 14474.3 ± 16970.8 |  |  |
| Ages |  | < 0.001 | 0.026 |
| ≤ 35 | 3280.2 ± 5936.4 |  |  |
| 34-45 | 3842.6 ± 5061.2 |  |  |
| 45-55 | 4636.1 ± 6127.0 |  |  |
| 55-65 | 4766.4 ± 6300.5 |  |  |
| ≥ 65 | 5155.3 ± 6235.7 |  |  |
| Sex |  | < 0.001 | 0.214 |
| Male | 4903.1 ± 6845.3 |  |  |
| Female | 4363.7 ± 5172.3 |  |  |
| Comorbidities Score |  | < 0.001 |  |
| 0 | 3457.9 ± 4087.1 |  |  |
| 1 | 5034.0 ± 5940.1 |  |  |
| ≥ 2 | 6554.9 ± 8883.2 |  |  |
| Comorbidities |  |  |  |
| Angina | 4638.0 ± 6165.7 | 0.269 | 0.304 |
|  | 4908.7 ± 4951.4 |  |  |
| Myocardial infarction | 4548.0 ± 5947.4 | < 0.001 | < 0.001 |
|  | 9767.8 ± 10751.0 |  |  |
| Ischemic heart disease | 4578.0 ± 6025.7 | < 0.001 | < 0.001 |
|  | 7184.3 ± 8424.4 |  |  |
| Heart failure | 4622.4 ± 5938.2 | 0.021 | < 0.001 |
|  | 6897.1 ± 14981.1 |  |  |
| Cerebrovascular disease | 4579.7 ± 6037.8 | < 0.001 | < 0.001 |
|  | 5716.4 ± 7157.1 |  |  |
| Hypertension | 4548.4 ± 5831.7 | < 0.001 | < 0.001 |
|  | 5078.0 ± 7206.4 |  |  |
| Malignancy | 4386.5 ± 6020.6 | < 0.001 | < 0.001 |
|  | 5422.0 ± 6329.0 |  |  |
| Diabetes mellitus | 3966.1 ± 4494.1 | < 0.001 | < 0.001 |
|  | 7109.6 ± 9583.9 |  |  |
| Surgery (Operation) |  | < 0.001 | < 0.001 |
| Surgical treatment | 6587.2 ± 7285.9 |  |  |
| Medical treatment | 3548.4 ± 5018.6 |  |  |
| Intensive care units |  | < 0.001 | < 0.001 |
| ICU admission | 11787.2 ± 12036.4 |  |  |
| Ward admission | 3484.7 ± 3202.4 |  |  |
| Intensive care unit types |  | 0.001 | < 0.001 |
| Medical ICU | 16863.0 ± 15554.7 |  |  |
| Surgical ICU | 15625.6 ± 14619.4 |  |  |
| Neurological ICU | 14788.7 ± 13632.6 |  |  |
| Emergent ICU | 11166.8 ± 11158.3 |  |  |

Abbreviations: AKI, acute kidney injury; ICU, intensive care units

P^1^, ANOVA analysis between the stages of AKI. P^2^, ANCONA analysis between the stages of AKI. In the ANCOVA analysis, factors that demonstrated a significant impact on total medical costs, including the duration of admission, age, sex, body mass index, comorbidities, admission to an intensive care unit, and surgery, were included for adjustment
